# Is there any association between genetically predicted periodontitis and ischemic stroke?

**DOI:** 10.1038/s41432-023-00889-1

**Published:** 2023-05-10

**Authors:** Satish Kumar

**Affiliations:** Arizona School of Dentistry & Oral Health, A.T. Still University, Mesa, AZ USA

**Keywords:** Periodontitis, Periodontitis

## Abstract

**Design:**

Mendelian randomization study.

**Case selection:**

Using a Mendelian randomization framework, the causal relationship between periodontitis [chronic periodontitis (CP) and aggressive periodontitis (AgP)] and ischemic stroke and its subtypes [cardioembolic (CE) stroke, large artery atherosclerosis (LAA), and small vessel occlusion (SVO)], were studied.

**Data analysis:**

Data from three large databanks, namely, UK Biobank, genome-wide association study of European ancestry, and MEGASTROKE consortium of European ancestry were used to obtain genetic variant information of CP, AgP, and ischemic stroke, respectively. UK Biobank contributed 950 cases of CP and 455,398 controls. Genome-wide association study of European ancestry contributed 851 AgP cases and 6836 controls. MEGASTROKE consortium of European ancestry contributed 34,217 cases of ischemic stroke and its subtypes and 406,111 controls. Statistical tests including inverse variant weighted method and sensitivity analyses were performed to analyse the causal inference and to verify the strength of the results.

**Results:**

A total of 15 single nucleotide polymorphisms (SNPs) for CP was obtained as genetic instruments. No causal inference of CP on ischemic stroke was found. Among the ischemic stroke subtypes, with the exception of CE stroke, no significant causal inference of CP on LAA or SVO was found. A total of 9 single nucleotide polymorphisms (SNPs) for AgP was obtained as genetic instruments. No causal inference of AgP on ischemic stroke and its subtypes were found.

**Conclusions:**

Neither CP nor AgP was found to have a causal inference on ischemic stroke and most of its subtypes. A probable causal effect of CP on CE stroke was noted.

## A Commentary on

**Ma C, Wu M, Gao J** **et al**.

Periodontitis and stroke: A Mendelian randomization study. *Brain Behav*. 2023; 10.1002/brb3.2888.

**GRADE Rating:** 

## Commentary

The risk of ischemic stroke has been shown to be higher among the patients with periodontitis compared to those without periodontitis in numerous observational studies^1–3^. These studies indicate association but not causality^4^. The causal effect of periodontal disease in atherosclerotic cardiovascular diseases including stroke is controversial due to the major limitation of confounding factors^5^. Even after adjusting for confounding factors, study results may still bear the effects of residual confounding as well as other inherent biases in the observational study designs. Interventional studies to evaluate whether periodontal treatment would lower the risk of atherosclerotic cardiovascular diseases have not been able to provide sufficient evidence^4^.

To overcome the effects of confounding factors, researchers have started utilizing study design referred to as Mendelian randomization. Individuals randomly inherit or do not inherit a genetic variant. Mendelian randomization is a type of research study that uses these genetic variants to study possible causal relationships between risk factors (exposure) and disease (outcomes). Because genetic variants are fixed at conception, mendelian randomization studies reduce the effect caused by confounding factors and reverse causation that are typically seen in traditional observational studies. Mendelian randomization studies could be considered as interface studies between conventional observational studies and interventional studies^6,7^. Mendelian randomization study designs operate under three major assumptions—(1) the genetic variant is associated with the risk factor (relevance assumption); (2) there is no other confounder that exists between genetic variant and outcome (independence assumption); and (3) the genetic variant exerts its effect on the outcome through the effect of the risk factor only (exclusion restriction). Readers of mendelian randomization studies should ensure that these assumptions are valid. Other items to consider while appraising mendelian randomization study designs include justification for selection of SNPs, accounting for weak instrument bias when using two different samples, clinically meaningful effect size, consistency of association with previous studies, among others^7,8^.

In this study, the authors have used the mendelian randomization design to study the causal relationship between periodontitis (CP and AgP) and ischemic stroke and its subtypes (CE stroke, LAA, and SVO). They utilised large sample data from national and multinational databanks in Europe to select the genetic variants of risk factors of CP and AgP and for the outcome of stroke. They conducted statistical tests to ensure the robustness of their results including checking for heterogeneity and pleiotropy that explains effects of genetic variant on the outcome via alternate biological pathways. Despite these strengths, the study had some limitations. Due to the paucity of genetic variants, the authors increased the statistical threshold from the conventional *p* value <5 × 10^−8^ to <1 × 10^−5^ to select the SNPs. In addition, risk factors and outcome data were derived from different datasets. It is unclear if these approaches of selection of genetic variants could have been associated with introduction of potential confounders or shared risk factors and affected the causal inference. The authors found no causal inference between AgP and all subtypes of ischemic stroke. They also found no causal inference between CP and two subtypes of ischemic stroke, LAA and SVO. However, they found a causal inference between the combined genetic variants of CP and CE stroke (OR, 1.052; 95% CI, 1.002–1.104; *p* = 0.042). This must be interpreted with caution as the effect size was small (1.052). In addition, the lower end of the 95% confidence interval was very close to the null (1.002) and could be interpreted as being influenced by weak instrument or having unknown confounding effects. In other words, the main conclusion of the study on the causal association between CP and CE stroke is based on weak association and may not be clinically meaningful. In addition, the conclusion of this study is inconsistent with previous study using mendelian randomization and that these associations may not be accurate and could be attributed to shared risk factors such as smoking^9^. The genetic variant data was obtained from European databases and hence may not represent the entire global population. Additional mendelian randomization studies from large databases representing different populations across the globe is necessary to support or refute the findings of this study. The authors mainly focused their discussion on the causal association between CP and CE stroke. Perhaps the interesting finding of this study was that there was no causal inference between AgP and CP to most subtypes of stroke which contradicts the findings of most conventional observational studies. Perhaps, the results of positive association between periodontal disease and atherosclerotic cardiovascular disease from conventional observational studies could have been confounded by shared risk factors. Additional investigations that compare the estimates of the current and other mendelian randomization studies to the estimates of conventional observational studies investigating the association between periodontal disease and stroke. Lastly, as majority of the readers would be aware that AgP and CP have been combined into one diagnosis of periodontitis in the latest classification of periodontal diseases^10^. Future studies should also clarify the case definitions of periodontitis in such studies that include data from large databanks.

Practice points

- When studying causal association of two multifactorial, chronic and complex diseases such as periodontal diseases and cardiovascular diseases, it is important to use novel and better study designs such as Mendelian randomization design using large datasets to overcome the drawbacks of conventional observational studies and especially when conducting prospective interventional randomized trials is difficult or not feasible. However, in the quest for finding causal inference between periodontal disease and systemic diseases such as atherosclerotic cardiovascular diseases including stroke, the importance of preventing and treating periodontal disease for the sake of oral health should not be forgotten.
- There is a huge body of evidence in the literature that supports oral health is critical for overall health. Regardless of whether periodontal disease causes stroke or not, clinicians should prevent and treat periodontal disease for the sake of oral health and educate patients that there are several shared common risk factors for both diseases such as smoking and diabetes. Avoiding or managing these risk factors and adding routine oral health behaviors including proper diet, exercise, daily brushing and flossing would reduce the overall risk for both periodontal and systemic diseases.

## References

[1] Hsu PW, Shen YW, Syam S, Liang WM, Wu TN, Hsu JT (2022). Patients with periodontitis are at a higher risk of stroke: a Taiwanese cohort study. J Chin Med Assoc.

[2] Sumayin Ngamdu K, Mallawaarachchi I, Dunipace EA, Chuang LH, Jafri SH, Shah NR (2022). Association between periodontal disease and cardiovascular disease (from the NHANES). Am J Cardiol.

[3] Lee YT, Tsai CF, Yen YC, Huang LK, Chao SP, Hu LY (2022). Periodontitis is a potential risk factor for transient ischemic attack and minor ischemic stroke in young adults: a nationwide population-based cohort study. J Periodontol.

[4] Lockhart PB, Bolger AF, Papapanou PN, Osinbowale O, Trevisan M, Levison ME (2012). Periodontal disease and atherosclerotic vascular disease: does the evidence support an independent association? a scientific statement from the American Heart Association. Circulation..

[5] Niederman R, Weyant R (2012). Periodontal disease, cardiovascular disease, the American Heart Association, the American Academy of Periodontology, and the rooster syndrome. Evid Based Dent.

[6] Emdin CA, Khera AV, Kathiresan S (2017). Mendelian randomization. JAMA.

[7] Davies NM, Holmes MV, Davey Smith G (2018). Reading Mendelian randomisation studies: a guide, glossary, and checklist for clinicians. BMJ..

[8] Skrivankova VW, Richmond RC, Woolf BAR, Yarmolinsky J, Davies NM, Swanson SA (2021). Strengthening the reporting of observational studies in epidemiology using Mendelian randomization: the STROBE-MR statement. JAMA..

[9] Zhou M, Dong J, Zha L, Liao Y (2021). Causal association between periodontal diseases and cardiovascular diseases. Genes (Basel).

[10] Caton JG, Armitage G, Berglundh T, Chapple ILC, Jepsen S, Kornman KS (2018). A new classification scheme for periodontal and peri-implant diseases and conditions - Introduction and key changes from the 1999 classification. J Clin Periodontol.

